# Serum dihydroxyacetone kinase peptide m/z 520.3 as predictor of disease severity in patients with compensated chronic hepatitis B

**DOI:** 10.1186/1479-5876-11-234

**Published:** 2013-09-27

**Authors:** Ming-Yi Xu, Xiao-Fang Jia, Ying Qu, Rui-Dan Zheng, Zheng-Hong Yuan, Hong-Lei Weng, Steven Dooley, Xing-Peng Wang, Li-Jun Zhang, Lun-Gen Lu

**Affiliations:** 1 Department of Gastroenterology, Shanghai First People's Hospital, Shanghai Jiaotong University School of Medicine, No. 100, Haining Road, Shanghai 200080, China; 2 Shanghai Public Health Clinical Center, Fudan University, Shanghai 201508, China; 3 Research and Therapy Center for Liver Diseases, Southeast Hospital, Zhangzhou, Fujian Province 363000, China; 4 Molecular Hepatology-Alcohol Associated Diseases, II. Medical Clinic Faculty of Medicine at Mannheim, University of Heidelberg, Mannheim 68167, Germany

**Keywords:** Peptidome, Dihydroxyacetone kinase, Chronic hepatitis B, Multiple reaction monitoring, Liquid chromatography combined with tandem mass spectrometry

## Abstract

**Background & aim:**

Due to known limitations of liver biopsy, reliable non-invasive serum biomarkers for chronic liver diseases are needed. We performed serum peptidomics for such investigation in compensated chronic hepatitis B (CHB) patients.

**Methods:**

Liquid chromatography combined with tandem mass spectrometry (LC-MS/MS) was used to identify differentially expressed peptides in sera from 40 CHB patients (20 with S0G0-S1G1 and 20 with S3G3-S4G4). Ion pair quantification from differentially expressed peptides in a validation set of sera from 86 CHB patients was done with multiple reaction monitoring (MRM).

**Results:**

21 differentially represented peptide peaks were found through LC-MS/MS. Ion pairs generated from eleven of these peptides (m/z < 800) were quantified by MRM. Summed peak area ratios of 6 ion pairs from peptide m/z 520.3 (176.1, 353.7, 459.8, 503.3, 351.3, 593.1), which was identified as dihydroxyacetone kinase (DAK) fragment, decreased from mild to advanced stages of fibrosis or inflammation. Area Under Receiver Operating Characteristic Curves (AUROCs) of five ion models discriminating fibrosis degrees were 0.871 ~ 0.915 (S2-4 versus S0-1) and 0.804 ~ 0.924 (S3-4 versus S0-2). AUROCs discriminating inflammation grades were 0.840 ~ 0.902 (G2-4 versus G0-1) and 0.787 ~ 0.888 (G3-4 versus G0-2). The diagnostic power of these models provides improved sensitivity and specificity for predicting disease progression as compared to aspartate aminotransferase to platelet ratio index (APRI), FIB-4, Forn’s index and serum DAK protein.

**Conclusions:**

The peptide fragment (m/z 520.3) of DAK is a promising biomarker to guide timing of antiviral treatment and to avoid liver biopsy in compensated CHB patients.

## Introduction

In China, chronic hepatitis B virus (HBV) infection is the most common cause of chronic liver disease. The number of hepatitis B surface antigen (HBsAg) carriers was estimated 9.09% of the population [1]. Accurate diagnosis of liver fibrosis and inflammation are crucial for the management of patients with chronic hepatitis B (CHB). Histological diagnosis via liver biopsy has long been the gold standard for assessing the degree of fibrosis and inflammation, but is an invasive procedure with inherent risks and sampling variability [2]. Non-invasive strategies based on blood tests, including clinical serological models [3-6], serological glycomics and proteomics [7-9], were recently successful in predicting advanced stages of fibrosis and inflammation in some settings. However, none of these tests can completely replace liver biopsy.

The peptidome has recently been recognized as a means for identifying novel biomarkers [10-15]. Circulating peptide fragments generated in body fluids or tissues can reflect biological events and provide critical information for clinical diagnosis [16,17]. Quantitation of peptide biomarkers in complex biological matrices, such as human serum, is a challenging task. A promising alternative method to antibody mediated approaches is mass spectrometry based multiple reaction monitoring (MRM) that allows accurately quantifying peptide.

Aim of this study was to find useful serum biomarkers indicating liver disease progression based on peptidomics in CHB patients. Liquid chromatography combined with tandem mass spectrometry (LC-MS/MS) was used to separate and identify differentially represented peptides in sera of CHB patients. Serum expression levels of such peptides were then quantitated by MRM.

## Materials and methods

The study included three parts: (I) a discovery study; (II) a validation study and (III) a diagnosis study.

### Patients

126 CHB patients that were HBsAg positive for at least 6 months were enrolled from Shanghai First People’s Hospital between 2008 and 2010. Patients were excluded, when they were co-infected with HIV or HCV, consumed >30 g alcohol per day, had received antiviral treatment, or without availability of sufficient liver biopsy tissue (a minimum length of 1.0 cm of the liver biopsy and at least 6 portal tracts included were the inclusion criteria). All CHB patients included in our study were compensated and end-stage liver disease was excluded. The study was approved by the Ethics Committee of Shanghai First People’s Hospital and informed consent was obtained from each patient. All patients received percutaneous liver biopsy. Fibrotic stage and inflammational grade of tissue samples were classified according to the Scheuer classification [18,19].

### Serum peptide extraction

Peripheral blood was taken before breakfast, collected in tubes containing EDTA-anticoagulant, and stored at -80°C until use. Serum peptides were extracted as described [20,21]; briefly, proteins were precipitated from serum by rapid addition of two volumes acetonitrile containing 0.1% trifluoroacetic acid, and immediately mixed by vortexing. Samples were incubated at 4°C for 30 min and centrifuged at 12,000 rpm for 30 min twice. The supernatant was collected and dried by vacuum centrifugation (Labconco Corporation, Kansas City, USA).

### Peptide profiling or identification by ion trap mass spectrometry

Peptide samples were re-lysed in 50 μl 2% acetonitrile (ACN), 0.1% formic acid (FA) and centrifuged. 15 μl of the solution were separated by use of Ultimate 3,000 instruments (LC Packings, Dionex, USA) through a C-18 reversed-phase nanocolumn (75 μm id × 15 cm length, 3 μm, PepMapTM) with a linear gradient from 4% to 48% acetonitrile and 0.1% formic acid for 60 minutes, after a desalting step across a C-18 precolumn (300 μm id × 5 mm, 5 μm, PepMapTM). Peptides eluted from the reversed-phase nanocolumn were on line injected by a PicoTip emitter nanospray needle (New Objective, Woburn) for real-time ionization and peptide fragmentation on an Esquire HCT ion-trap mass spectrometer (Bruker-Daltonics, Bremen, Germany). MS acquisition parameters were set as follows: ESI-Ion-trap MS, positive mode, time range: 600–4,500 seconds, scanning range: m/z of 100–2,800, target mass: 900 m/z. Each sample was analyzed three times, two for peptide profiling and one for peptide identification. For peptide profiling, only MS scanning was performed. For differentially represented peptide identification, autoMS(n) was performed. Before the experiment, HCT mass spectrometry was calibrated by tune mix.

For peptide profiling, MS scan data were further analyzed with ProfileAnalysis software (Bruker Daltonics, Germany) for MS-*T*-Test Model analysis. Bucketing parameters were as follows: Δ m/z =1 Da, Δ RT = 60 seconds. Each data set was normalized to the sum of bucked values. Criteria of differentially expressed peaks were *p* value < 0.05, with m/z >300, retention time >10 minutes, fold change >1.5 and detection in more than 80% of samples. MS/MS data were analyzed through calculation of scored peak intensity (SPI). The peptide peak, which had a charge of +2 with a score threshold of at least 10, or a charge of +1 or +3 with a score threshold of at least 13, was considered a good hit, if it also had a SPI of at least 70. Furthermore, all identified peptides will be checked to have *p* values less than 0.05 (*p* < 0.05) [22].

To identify differentially represented peptides, autoMS(n) data were processed with DataAnalysis software (Bruker Daltonics, Germany) to generate a Mascot generic format file (MGF, Matrix Science Inc.). The MGF file was used to search against the IPI_human database by Mascot. Peptides with a score >15 (*p* < 0.05) were considered significant. Identification of differentially represented peptides was achieved with a laboratory-created program to compare MS scan profile analysis with autoMS(n) scan peptide database searching results. Peptide peaks that matched with peptide identification results of the same retention time and m/z were considered successfully identified.

We applied bio-informatics software (Bruker Profile Analysis and IPI_human protein database) for spectra analyses for improved detection of candidate MS peaks. To confirm that the sequence of each peptide fragment reported in this study was not present in more than one protein, all sequences were proven in the blastp database (http://www.ncbi.nlm.nih.gov/blast/).

### Quantitative peptide scanning by MRM

MRM was performed with a combination of Shimadzu HPLC and Applied Bio-systems API3200 mass spectrometry. Quantitative scanning was based on parameters from the peptide ELNNALQNLARTI (ESAT-6), which represents amino acids 64-76 of early secreted antigenic target protein 6 from mycobacterium tuberculosis, which is absent in our samples. 45 ml volume of extracted serum peptide sample was chromatographed on an Acclaim PepMap C-18 column with a total flow of 0.06 ml/min. The precursor ion was isolated with a mass window of 2.0 m/z units and fragmented (collision energy = 35%, activation time = 30 ms at Q = 0.25); the resulting fragment ion was scanned in profile mode with a mass window of 2.0 m/z unit. According to API3200 detection sensitivity, we chose target peptides with m/z values <800 and product ions with relatively small m/z values for MRM analysis. At least 2 transitions and the product ions were monitored for target and reference peptides. Signature ion pairs for each target peptide were selected based on their uniqueness and chemical stability. Analyst software was used to extract and integrate transition ion peak areas for each target peptide.

### Study focus on peptide m/z 520.3

#### Verification of the identity of the m/z 520.3 by Q TOF

To verify the peptide m/z 520.3, we synthesized the peptide LLSKLSVLLLEKMG + Oxidation (M) by Shanghai Qiangyao Biotech Co.,Ltd., Shanghai, China. The synthesized peptide and serum peptide extractions from clinical samples were analyzed with mass spectrometry Quadrupole orthogonal TOF (Q TOF) (QSTARXL, Applied Biosystems, USA), with high resolution and mass accuracy. Samples were directly injected into MS through a nano spray needle. The instrument was operated in the positive ion mode. The initial MS scan utilized an m/z range of 400–2,000, with three precursors selected for interrogation from each MS survey scan. Precursor selection was based on ion intensity (peptide signal intensity above ten counts), charge state, and based on whether the precursor was previously selected for interrogation (dynamic exclusion). External calibration was carried out prior to analysis using horse myoglobin digest peptides.

#### Verification of the expression of peptide m/z 520.3

The synthesized peptide LLSKLSVLLLEKMG + Oxidation (M) was used to assess the method for quantifying m/z 520.3. The synthetic peptide from ESAT-6 was used as internal standard. In order to decrease side effects from endogenous peptides, 5 ml plasma from a person containing m/z 520.3 near the low limit of quantification (LLOQ) was used as blank in the experiments described in sections Accuracy and precision analysis and Stability analysis of serum peptide m/z 520.3 by MRM below. Values for cross-contamination of the blank were subtracted from values for the spiked standard samples.

##### Accuracy and precision analysis

Six ion pairs of m/z 520.3 were analyzed. Six calibration standards with concentrations of 6.25, 12.5, 25, 50, 100 and 200 ng/μl were used to proof the MRM methods. Three quality controls (QC) with concentrations of 7.5, 75 and 160 ng/μl, were analyzed to proof accuracy and precision. Each QC was prepared in parallel experiments.

##### Stability analysis of serum peptide m/z 520.3 by MRM

Stability studies were conducted using mixed standards with a synthesized peptide of 1 ng/μl and an internal standard (IS) of 0.4 ng/μl. Stability of analytes at 4°C was assessed at intervals of 8, 16 and 24 h in triplicate aliquots. To proof long-term stability during storage, samples frozen for up to 2 months were tested at intervals of 20, 40 and 60 days. Sera from 6 healthy controls or their sera spiked with 50 ng/μl of synthetic peptide were used. Internal standard was added prior to sample preparation at each scheduled time, and the stability for each sample was determined through 6 ion pairs.

#### Quantification of peptide m/z 520.3 and others in serum samples

For quantification of m/z 520.3 and other peptides, 11 ion pairs and then 6 ion pairs of m/z 520.3 were scanned. Summed peak areas of product ion pairs were calculated. We also calculated the summed peak areas ratio (SPAR) of ion pairs generated from target and internal standard peptide (ESAT-6). SPAR values of groups were compared with Kruskal-Wallis non-parametric tests.

### Establishment of diagnostic models

Ion pairs of the marker peptide were used to establish a diagnostic model. SPAR values were used as diagnostic values. Aspartate aminotransferase-to-platelet ratio index (APRI), FIB-4 and Forn’s index were calculated according to the following formulas: [23-25] APRI = [AST (/ULN)/PLT (10^9^/L)] × 100, FIB − 4 = [age (yr) × AST (U/L)]/{[PLT (10^9^/L)] × (ALT (U/L)]1/2}, Forn ’ s index = 7.811 − 3.131 × ln [PLT (10^9^/L)] + 0.781 × ln [GGT (U/L)] + 3.467 × ln [age (yr)] − 0.014 × [cholesterol (g/L)]. AUROC analysis was used to calculate diagnostic sensitivity and specificity.

### ELISA

Serum DAK protein was examined by an ELISA Kit (Assay Biotechnology Company, Inc. California, USA). The DAK antibody detects endogenous levels of total DAK protein. All 126 serum samples from enrolled CHB patients were analyzed.

### Statistical analysis

The *χ*^2^ test was used for testing categorical variables; the Student’s *t*-test was used for continuous variables; univariate analysis with SPSS 19.0 (SPSS Inc, Chicago) was used. ROC curve analysis was applied to determine the cutoff value for peptide levels by the 0.1-criterion, and the areas under curves (AUCs) were calculated. P < 0.05 was considered statistically significant.

## Results

### Discovery study

In the discovery study, LC-MS/MS was performed with serum samples from 40 CHB patients classified according to the degree of fibrosis and inflammation into Early Disease (ED, including 10 patients with S0G0 and 10 patients with S1G1) or Advanced Disease (AD, 10 patients with S3G3 and 10 patients with S4G4). Their characteristics are shown in Additional file 1: Table S1. In total, 296 differential peptide peaks were evaluated by LC-MS/MS and ProfileAnalysis software. 21 independent peaks with significantly different mean SPI values between two groups were identified (Table 1). We provide the typical MS maps in m/z range of 500-800 of different stages (S0, G0, S1, G1, S3, G3, S4, G4, Additional file 2: Figure S1).

**Table 1:** Differential peptide identified in LC-MS/MS analysis

	**PA**	**PA**	** Mean SPI (E + 05)**		***p *value**	**Fold**	**Differential peptide sequence**	**IP**	**GENE**	**Protein Information**
	**Time(s)**	**m/z**	**MF**	**SF**		**MF/SF**		**Tax Id = 9606**		
**1**	2670	545.5	62.52	15.70	0.034	-3.22	AAGFLLMYST + Oxidation (M)	IPI00644710	SLC35D2	Isoform2 of UDP-N-acetylglucosamine
**2**	2910	427.5	16.12	4.55	0.045	2.76	PTNFAPVINHVA	IPI00334276	CPNE8	cDNA FLJ25727 fis,clone TST05479
**3**	**3150**	**520.3**	**3.26**	**6.83**	**0.049**	**2.14**	**LLSKLSVLLLEKMG + Oxidation (M)**	**IPI00551024**	**DAK**	**ATP-dependent dihydroxyacetone kinase**
**4**	2790	414.5	4.97	2.31	0.003	1.68	GPMNMNMGMNM + Oxidation (M)	IPI00030652	ZIC2	Zinc finger protein ZIC 2
**5**	3390	640.5	22.93	7.92	0.025	2.26	AANSQPQPPRES	IPI00221255	MYLK	Isoform 2 of Myosin light chain kinase
**6**	3450	550.5	14.43	4.19	0.023	2.68	AAINKMCVFS + Oxidation (M)	IPI00922072	-	cDNA FLJ52618
**7**	4230	816.5	19.91	9.41	0.048	1.83	STPPITSSITPTDTMTSMRTTTS +2 Oxidation (M)	IPI00386766	MUC3A	Isoform 2 of Mucin-3A
**8**	2130	327.5	1.73	5.55	0.048	-4.11	MEGNKTWI	IPI00455038	OR2A14	Olfactory receptor 2A14
**9**	2070	787.5	6.29	3.16	0.046	1.55	ELGLEMTAGFGLGGLRLTALQAQ + Oxidation (M)	IPI00063762	HPDL 4	Hydroxyphenylpyruvate dioxygenase-like protein
**10**	2910	1013.5	4.04	7.45	0.044	-2.63	NYEESIKMPINEPAPGKKKSQIQEYV + Oxidation (M)	IPI00218297	HPD 4	Hydroxyphenylpyruvate dioxygenase
**11**	3030	869.5	40.06	20.43	0.049	1.53	LPCTESSSSMPGLGMVPPPPPPLPGM + 2 Oxidation (M)	IPI00742944	FMN2	FMN2 195 kDa protein
**12**	3390	881.5	10.90	4.28	0.031	1.99	MTCTYVCVCVYMYVCIYIYMY + Oxidation (M)	IPI00943356	-	Putative uncharacterized protein (Fragment)
**13**	3810	766.5	6.08	2.44	0.020	1.75	GTRRRCPCAPRSGLPGRRSVD	IPI00936509	-	LOC100287493; hypothetical protein
**14**	4230	839.5	10.26	4.01	0.035	2.21	EEPPPPSS	IPI00386642	MEF2B	LOC729991 Isoform 1 of UPF0402 protein
**15**	2070	682.5	24.48	51.19	0.048	-2.68	DQGLYHCIATEN	IPI00019209	-	SEMA3C cDNA FLJ55486
**16**	3270	1129.5	7.23	5.98	0.021	-5.32	CLSYMALLRLPKKRGTFIEFRNGMLNISP + Oxidation (M)	IPI00294903	PMM1	Phosphomannomutase 1
**17**	3030	981.5	5.35	8.00	0.024	-1.92	NVARMLALALAESAQQAST + Oxidation (M)	IPI00787743	ARHGAP32	Isoform 1 of Rho/Cdc42/Rac GTPase-activating protein
**18**	2790	757.5	71.50	30.99	0.049	1.8	DEPVSGELVSVAHALSLPAESY	IPI00107104	CXorf26	UPF0368 protein Cxorf26
**19**	3150	1142.5	5.49	2.23	0.045	1.91	LTVLWYGVVHTSALVRCTAARMFELTLRGM + 2 Oxidation (M)	IPI00101291	KIAA1468	KIAA1468, isoform CRA
**20**	3450	901.5	14.39	7.12	0.008	1.58	MPKTWISWAEIRSHTSSLSMSHP +2 Oxidation (M)	IPI00643635	C20orf57	DUSP15 Dual specificity phosphatase 15
**21**	3930	857.5	15.45	5.17	0.036	2.33	AVNWVARSLYWTHTGTEHIEVT	IPI00018681	LRP5L	Isoform 2 of Low-density lipoprotein receptor-related protein 5-like protein

### Validation study

#### Peptide m/z 520.3 was singled out by MRM analysis

**Figure 1:**
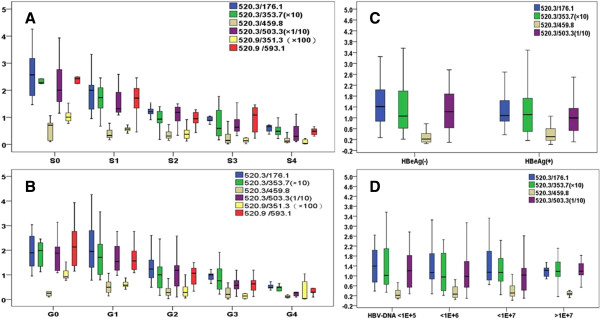
**SPAR values of 6 ion pairs from peptide m/z 520.3.** Box plot figure shows values of 4-6 ion pairs generated from peptide m/z 520.3 in 86 serum samples from CHB patients. **A**: Patients were classified in 5 groups according to fibrosis stages (S0: n = 13, S1: n = 22, S2: n = 20, S3: n = 16, S4: n = 15). SPAR values of 6 ions of m/z 520.3 display a statistically significant decrease in groups S1 ~ S4 as compared to S0. Different colors represent different ions (blue = 520.3/176.1, green = 520.3/353.7, brown = 520.3/459.8, purple = 520.3/503.3, yellow = 520.3/351.3, red = 520.3/593.1). **B**: SPAR values decrease along with liver inflammation severity in the 5 groups classified by inflammation grades. **C**: SPAR values have no statistically significant difference between groups classified by HBeAg carrier status. **D**: SPAR values have no statistically significant difference among groups classified by HBV-DNA levels (HBV-DNA: 10^4^ ~ 10^5^; 10^5^ ~ 10^6^; 10^6^ ~ 10^7^; ≥10^7^).

In the validation study, MRM was conducted with serum samples from 86 CHB patients. All patients could be sorted out to 4 classifications regarding fibrotic degree (S0-S4), inflammatory grade (G0-G4), HBeAg carrier (HBeAg + or HBeAg-), and HBV-DNA level. Clinical characteristics are shown in Additional file 1: Table S1. Selection of target peptides was based on the 21 peptides found differentially represented in the discovery study. 11 of those were selected as target peptides (m/z <800). These generated 2 ~ 4 ion pairs each, resulting in a total of 38 product ion pairs that were exploited and confirmed as stable and reliable. Selected transitions were verified by comparing the relative signal area. In order to maximize the number of data points across the peaks, in the final assay one single transition was chosen from each target peptide (Additional file 3: Table S2: ion pairs marked in red colour were analyzed). For the above-selected 11 ion pairs, SPAR values were calculated and statistically analyzed among subgroups (S0-S4 and G0-G4). Only SPAR values of 520.3/176.1 were significantly different among S1-S4 as compared to S0 and G1-G4 as compared to G0 (*p* < 0.05; Figure 1A/B), whereas those of the 10 other ions were not (we show data of additional 2 ions in Additional file 4: Figure S2). Thus, we focused on finding out whether serum peptide m/z 520.3 levels correlates with disease progression in CHB patients.

#### Accuracy, precision and stability of peptide m/z 520.3 in MRM methodology

The correlation coefficients (r) of calibration curves were >0.99 for 735.5/389.3 for ESAT-6 and six ions of m/z 520.3, as determined by linear analysis. They had an accuracy of 100 ± 20% in the quantification range (Additional file 5: Figure S3).

Intra-day accuracy and precision varied from 92.1% to 113.8% and from 2.1% to 11.0%, respectively. Inter-day accuracy and precision varied from 95.3% to 107.4% and from 6.1% to 10.9%, respectively (Additional file 6: Table S3). These values are well within acceptance criteria, as recommended by the China FDA guidelines (2005, China Pharmacopoeia).

Relative errors (RE%) for 6 ions of m/z 520.3 were 2.6 ~ 15.3% at intervals of 8 ~ 24 h and at 4°C. Ratios of standard and average values (STD/AVE) were 0.120 ~ 0.199 at intervals of 20 ~ 60 days at -80°C, indicating acceptable sample storage short or long term stability (Additional file 7: Table S4).

#### Quantification and identification of peptide m/z 520.3

**Figure 2:**
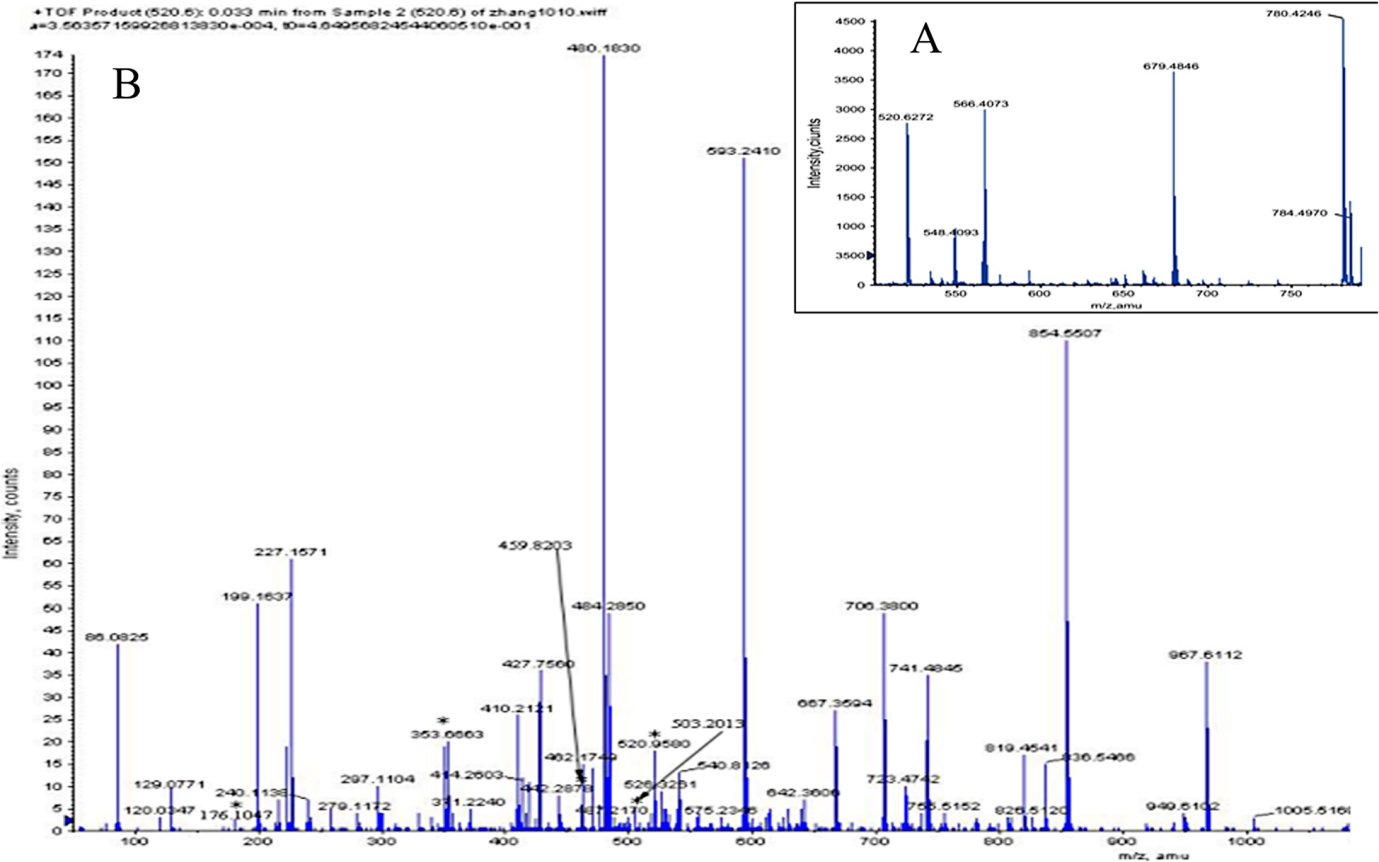
**Mass spectrometry and tandem mass spectrometry of peptide with m/z 520.3. A**: Mass spectrometry (MS) in m/z range from 500 to 800 including target peptide peak with m/z of 520.3 (520.6272). **B**: Tandem mass spectrometry (MS/MS) in m/z range from 0 to 1100 including peptide with m/z of 520.3 (520.9580). Four transition spectra with double charge (520.3/176.1; 520.3/353.7; 520.3/459.8; 520.3/503.3) are also marked in the figure.

Peptide m/z 520.3 displayed 4 fragment ions with double charges (520.3/176.1; 520.3/353.7; 520.3/459.8; 520.3/503.3) and 2 with single charges (520.3/351.3; 520.3/593.1) (Figure 2).

**Figure 3:**
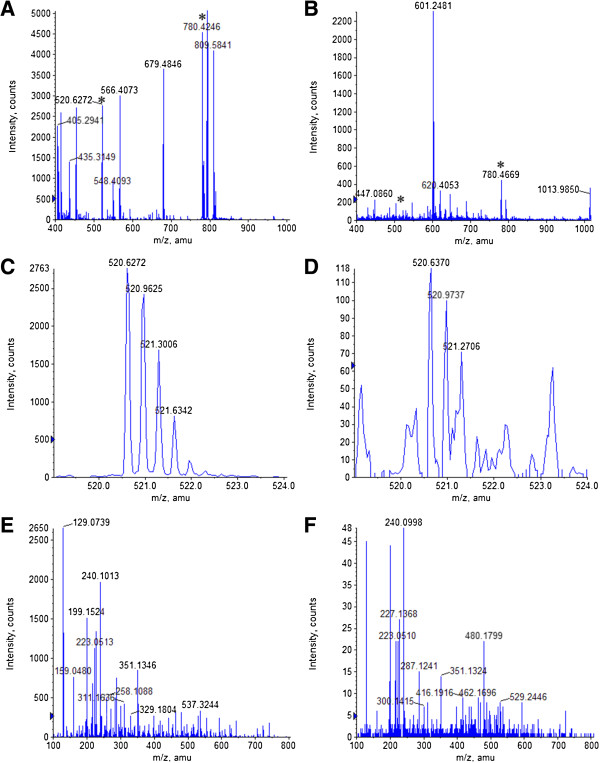
**Spectrogram of synthesized DAK peptide and peptide extracted from patient serum through Qstar mass spectrometry. A** to **C** represent MS profiles of synthetic peptide. **D** to **F** represent MS profiles of serum extracted peptide from a patient with fibrosis stage S2 (Sample No. 62). **A** and **D** show the ms profile. **B** and **E** are a partial enlargement of 520.3 (also 520.6). **C** and **F** are MS-MS of 520.3 (also 520.6), partial magnification and MS-MS of 50 ng/μl synthetic DAK peptide.

We identified the peptide sequence with m/z of 520.3 as LLSKLSVLLLEKMG + Oxidation (M) by Mascot IPI_human database searching. Its accession number in the IPI database is IP100551024, and the gene symbol is dihydroxyacetone kinase (DAK). In order to verify the peptide m/z 520.3, we synthesized the peptide LLSKLSVLLLEKMG + Oxidation (M). MS and MS/MS profiles from the synthesized peptide and the peptide extracted from a clinical sample with fibrosis stage S2G1 (Sample No. 64) were generated by high resolution mass spectrometry Q TOF. The exact m/z of the LLSKLSVLLLEKMG + Oxidation (M) peptide was 520.6 (Figure 3A and D), and similar MS/MS profiles (Figure 3C and F) of the precursor ion 520.6 were obtained with the synthesized peptide and the peptide extracted from patient serum. As can be seen from partial enlargement, the characters of +3 ions were obvious (Figure 3B and E). MS-MS values were identical with calculated ones (data not shown), and specific precursor and fragment ions were clearly detectable (Additional file 5: Figure S3). Furthermore, MS-MS profiles for 520.6 and 520.3 were very similar (data not shown). These results confirm peptide identification data, suggesting that the differentially represented peptide 520.3 detected in HCT and API3200 mass spectrometry is definitely LLSKLSVLLLEKMG + Oxidation (M).

In order to confirm the presence of 520.3, we detected 6 ion pairs in each of 86 samples. SPAR values statistically decreased among groups from mild to severe fibrotic stages. Ion representation levels were statistically different in groups S1-S4, as compared to S0 (Figure 1A). SPAR values of 6 ion pairs as well gradually decreased mild to severe inflammatory grades G0 to G4 (Figure 1B). No statistically significant difference was found among groups classified by HBeAg carrier status or HBV-DNA levels (Figure 1C/D). Our results suggest that serum peptide m/z 520.3 levels reflect disease progression since its detection levels consistently rise with the degree of liver fibrosis and inflammation.

### Diagnosis study

**Figure 4:**
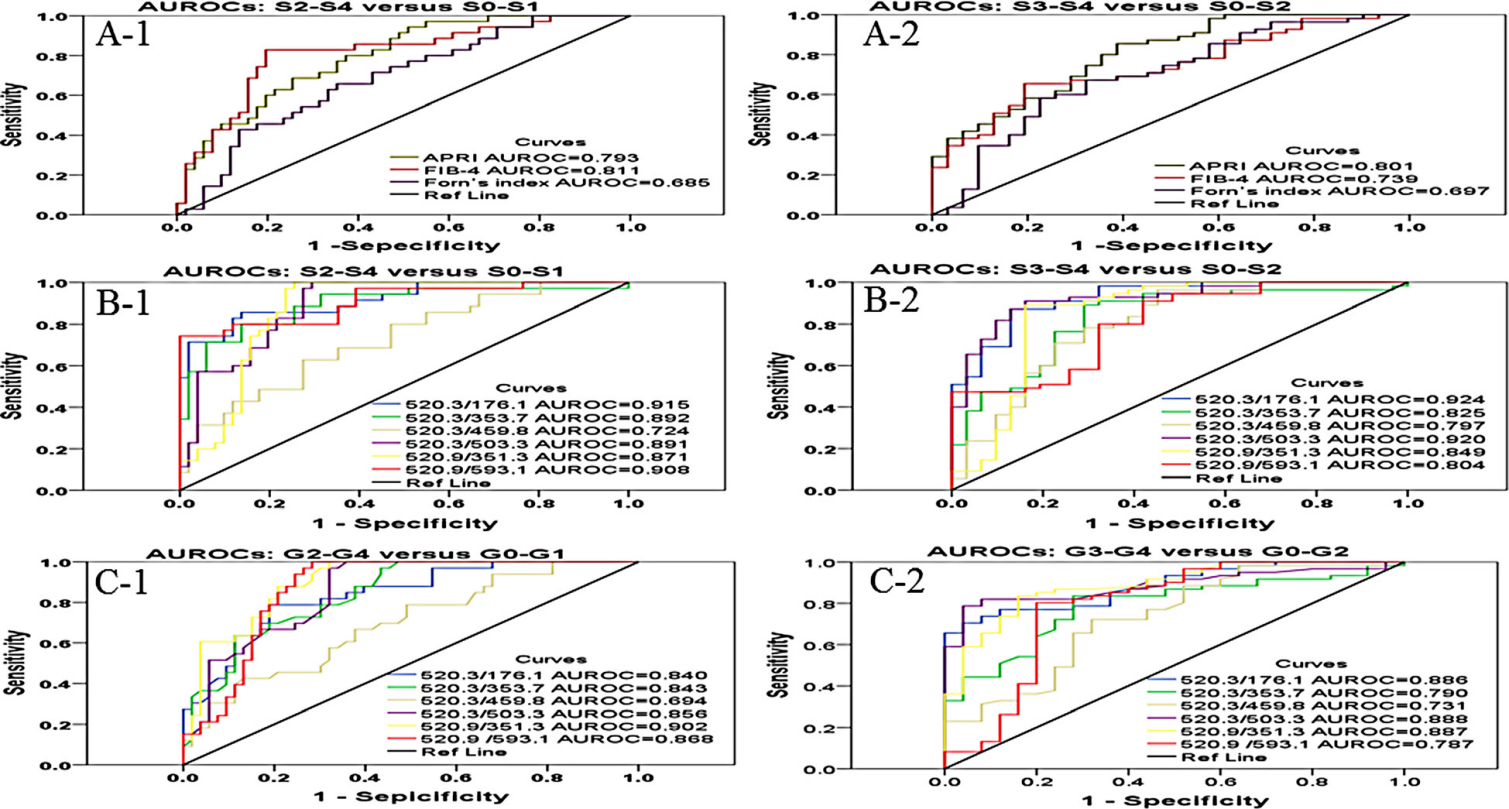
**AUROCs of ion pairs generated from peptide m/z 520.3.** Six models established by ions of peptide m/z 520.3 (176.1, 353.7, 459.8, 503.3, 351.3 and 593.1) and three serological models currently in clinical use (APRI, FIB-4, Forn’s Index) were evaluated for their diagnostic potential by AUROC. **A-1**: AUROCs of APRI, FIB-4, Forn’s Indices are 0.724 ~ 0.876 (S2-4 versus S0-1). **A-2**: AUROCs of APRI, FIB-4, Forn’s Indices are 0.758 ~ 0.839 (S3-4 versus S0-2). **B-1**: AUROCs of 6 ions from 520.3 models are 0.724 ~ 0.940 (S2-4 versus S0-1). **B-2**: AUROCs of 6 ions from 520.3 models are 0.883 ~ 0.937 (S3-4 versus S0-2). **C-1**: AUROCs of 4 ions from 520.3 models are 0.694 ~ 0.856 (G2-4 versus G0-1). **C-2**: AUROCs of 4 ions from 520.3 models are 0.731 ~ 0.888 (G3-4 versus G0-2).

We established 6 models with ions of peptide m/z 520.3 (176.1, 353.7, 459.8, 503.3, 351.3, 593.1). Diagnostic values were the respective SPAR values. Values of AUROC, 95% CI, cut-off value, sensitivity and specificity are shown in Table 2. AUROCs of 6 ions models (176.1, 353.7, 459.8, 503.3, 351.3, 593.1) distinguishing fibrosis stages S2-S4 from S0-S1 were 0.915, 0.892, 0.724, 0.891, 0.871 and 0.908, respectively (Figure 4B-1). AUROCs distinguishing S3-S4 from S0-S2 were 0.924, 0.825, 0.797, 0.920, 0.849 and 0.804, respectively (Figure 4B-2). AUROCs of APRI, FIB-4 and Forn’s index were 0.793, 0.811 and 0.685 to distinguish S2-S4 from S0-S1, and 0.801, 0.739 and 0.697 to distinguish S3-S4 from S0-S2 (Figure 4A). Ion models of m/z 520.3, with one exception (520.3/459.8), had a better diagnostic power to identify liver fibrosis stages, as compared to the above clinical serological models (APRI, FIB-4 and Forn’s index). In AUROC comparisons between ions of peptide m/z 520.3, APRI, FIB-4 and Forn’s index, *t*-test and P-value are provided in Table 3 to indicate statistical differences. Most sensitivities and 1-specificities of peptide ions 520.3/353.7, 520.3/459.8 and 520.3/503.3 were significantly superior to APRI, FIB-4 and Forn’s index.

**Table 2:** Diagnostic values of established ions of peptide m/z 520.3 models and others

	**S2-S4 versus S0-S1**						**S3-S4 versus S0-S2**					
	**AUROC**	** 95% CI**		**Cut-off value**	**Sensitivity**	**Specificity**	**AUROC**	** 95% CI**		**Cut-off value**	**Sensitivity**	**Specificity**
520.3/176.1	0.915	0.855	0.976	1.401	0.886	0.902	0.924	0.936	0.948	1.287	0.868	0.98
520.3/353.7	0.892	0.818	0.967	0.161	0.829	0.922	0.825	0.855	0.929	0.129	0.732	0.919
520.3/459.8	0.724	0.617	0.832	0.368	0.629	0.725	0.797	0.692	0.902	0.337	0.692	0.902
520.3/503.3	0.891	0.824	0.957	12.751	0.851	0.865	0.92	0.916	0.965	9.050	0.862	0.979
520.3/351.3	0.871	0.793	0.949	43.85	0.882	0.852	0.849	0.954	1.000	28.91	0.745	0.954
520.3/593.1	0.908	0.842	0.974	1.769	0.829	0.903	0.804	0.795	0.947	1.487	0.71	0.899
FIB-4	0.793	0.7	0.885	222.70	0.757	0.801	0.801	0.705	0.896	408.77	0.836	0.645
Forn’s Index	0.811	0.715	0.906	-317.1	0.857	0.686	0.739	0.635	0.843	-416.01	0.673	0.742
APRI	0.685	0.573	0.798	33.79	0.771	0.643	0.697	0.577	0.816	59.94	0.655	0.677
	**G2-G4 versus G0-G1**						**G3-G4 versus G0-G2**					
	**AUROC**	** 95% CI**		**Cut-off value**	**Sensitivity**	**Specificity**	**AUROC**	** 95% CI**		**Cut-off value**	**Sensitivity**	**Specificity**
520.3/176.1	0.84	0.756	0.924	1.301	0.788	0.811	0.756	0.818	0.953	1.186	0.705	0.760
520.3/353.7	0.843	0.763	0.923	0.184	0.870	0.666	0.763	0.69	0.889	0.179	0.806	0.720
520.3/459.8	0.694	0.581	0.807	0.335	0.788	0.509	0.581	0.61	0.852	0.235	0.621	0.680
520.3/503.3	0.856	0.779	0.933	10.375	0.910	0.679	0.779	0.819	0.956	10.375	0.887	0.910
520.3/351.3	0.902	0.839	0.964	43.60	0.911	0.851	0.839	0.813	0.96	41.82	0.901	0.842
520.3/593.1	0.868	0.792	0.945	1.653	0.876	0.831	0.792	0.66	0.914	1.644	0.874	0.792

**Table 3:** AUROCs comparison between ions of peptide m/z 520.3, APRI, FIB-4 and Forn’s index

		**1-Specificity S0-2 vs S3-4**								**Sensitivity S0-2 vs S3-4**							
		**Mean**	**Std. deviation**	**Std. error mean**	**95% CI of the difference**		**t**	**df**	**P-value**	**Mean**	**Std. deviation**	**Std. error mean**	**95% CI of the difference**		**t**	**df**	**P-value**
APRI	520.3/176.1	(0.01)	0.48	0.05	(0.12)	0.10	(0.16)	76.00	0.87	0.09	0.60	0.07	(0.04)	0.23	1.34	76.00	0.19
	520.3/353.7	0.18	0.46	0.06	0.06	0.29	3.07	63.00	0.00	0.18	0.56	0.07	0.04	0.32	2.62	63.00	0.01
	520.3/459.8	0.16	0.41	0.05	0.06	0.27	3.18	62.00	0.00	0.15	0.57	0.07	0.01	0.30	2.15	62.00	0.04
	520.3/503.3	0.08	0.43	0.05	(0.03)	0.19	1.54	60.00	0.13	0.18	0.57	0.07	0.04	0.33	2.53	60.00	0.01
	520.9/351.3	(0.03)	0.51	0.05	(0.14)	0.08	(0.56)	86.00	0.57	0.02	0.64	0.07	(0.12)	0.15	0.25	86.00	0.80
	520.9/593.1	0.07	0.48	0.06	(0.04)	0.18	1.30	76.00	0.20	0.06	0.59	0.07	(0.08)	0.19	0.86	76.00	0.39
FIB-4	520.3/176.1	(0.05)	0.52	0.06	(0.17)	0.07	(0.84)	76.00	0.40	0.11	0.58	0.07	(0.02)	0.25	1.74	76.00	0.09
	520.3/353.7	0.15	0.49	0.06	0.03	0.27	2.42	63.00	0.02	0.20	0.54	0.07	0.06	0.33	2.95	63.00	0.00
	520.3/459.8	0.14	0.44	0.06	0.03	0.25	2.49	62.00	0.02	0.17	0.55	0.07	0.03	0.31	2.44	62.00	0.02
	520.3/503.3	0.06	0.45	0.06	(0.05)	0.18	1.08	60.00	0.29	0.20	0.55	0.07	0.05	0.34	2.77	60.00	0.01
	520.9/351.3	(0.07)	0.53	0.06	(0.18)	0.04	(1.22)	86.00	0.23	0.04	0.62	0.07	(0.09)	0.17	0.59	86.00	0.56
	520.9/593.1	0.03	0.53	0.06	(0.09)	0.15	0.51	76.00	0.61	0.08	0.57	0.07	(0.05)	0.21	1.24	76.00	0.22
Forn’s	520.3/176.1	(0.08)	0.49	0.06	(0.19)	0.03	(1.42)	76.00	0.16	0.13	0.60	0.07	(0.00)	0.27	1.93	76.00	0.06
	520.3/353.7	0.11	0.47	0.06	(0.01)	0.22	1.83	63.00	0.04	0.22	0.55	0.07	0.08	0.36	3.21	63.00	0.00
	520.3/459.8	0.10	0.42	0.05	(0.01)	0.20	1.82	62.00	0.04	0.19	0.56	0.07	0.05	0.33	2.72	62.00	0.01
	520.3/503.3	0.02	0.43	0.06	(0.09)	0.13	0.32	60.00	0.75	0.22	0.57	0.07	0.08	0.37	3.06	60.00	0.00
	520.9/351.3	(0.10)	0.51	0.05	(0.20)	0.01	(1.77)	86.00	0.08	0.05	0.64	0.07	(0.08)	0.19	0.79	86.00	0.43
	520.9/593.1	0.00	0.50	0.06	(0.11)	0.11	0.03	76.00	0.98	0.10	0.59	0.07	(0.04)	0.23	1.45	76.00	0.15
		**1-Specificity S0-1 vs S2-4**								**Sensitivity S0-1 vs S2-4**							
		**Mean**	**Std. deviation**	**Std. error mean**	**95% CI of the difference**		**t**	**df**	**P-value**	**Mean**	**Std. deviation**	**Std. error mean**	**95% CI of the difference**		**t**	**df**	**P-value**
APRI	520.3/176.1	0.02	0.55	0.06	(0.11)	0.14	0.24	76.00	0.81	0.11	0.56	0.06	(0.01)	0.24	1.79	76.00	0.08
	520.3/353.7	0.16	0.52	0.06	0.03	0.29	2.50	63.00	0.02	0.21	0.53	0.07	0.07	0.34	3.12	63.00	0.00
	520.3/459.8	0.20	0.48	0.06	0.08	0.32	3.31	62.00	0.00	0.10	0.56	0.07	(0.04)	0.24	1.40	62.00	0.17
	520.3/503.3	0.12	0.47	0.06	0.00	0.25	2.05	60.00	0.04	0.18	0.58	0.07	0.03	0.33	2.47	60.00	0.02
	520.9/351.3	(0.03)	0.58	0.06	(0.15)	0.09	(0.51)	86.00	0.61	0.05	0.61	0.07	(0.08)	0.18	0.70	86.00	0.49
	520.9/593.1	0.03	0.55	0.06	(0.10)	0.15	0.42	76.00	0.68	0.12	0.56	0.06	(0.01)	0.24	1.82	76.00	0.07
FIB-4	520.3/176.1	0.02	0.56	0.06	(0.10)	0.15	0.36	76.00	0.72	0.10	0.54	0.06	(0.02)	0.23	1.66	76.00	0.10
	520.3/353.7	0.18	0.51	0.06	0.05	0.31	2.75	63.00	0.01	0.18	0.52	0.07	0.05	0.31	2.80	63.00	0.01
	520.3/459.8	0.22	0.48	0.06	0.10	0.34	3.59	62.00	0.00	0.07	0.56	0.07	(0.07)	0.21	1.05	62.00	0.30
	520.3/503.3	0.14	0.47	0.06	0.02	0.26	2.40	60.00	0.02	0.16	0.58	0.07	0.01	0.30	2.08	60.00	0.04
	520.9/351.3	(0.02)	0.59	0.06	(0.15)	0.10	(0.38)	86.00	0.70	0.04	0.59	0.06	(0.09)	0.16	0.55	86.00	0.58
	520.9/593.1	0.03	0.57	0.06	(0.09)	0.16	0.53	76.00	0.60	0.10	0.54	0.06	(0.02)	0.23	1.69	76.00	0.10
Forn’s	520.3/176.1	(0.03)	0.56	0.06	(0.16)	0.09	(0.53)	76.00	0.60	0.19	0.56	0.06	0.06	0.31	2.93	76.00	0.00
	520.3/353.7	0.11	0.53	0.07	(0.02)	0.24	1.67	63.00	0.10	0.28	0.51	0.06	0.15	0.41	4.40	63.00	0.00
	520.3/459.8	0.15	0.49	0.06	0.02	0.27	2.40	62.00	0.02	0.17	0.54	0.07	0.04	0.31	2.53	62.00	0.01
	520.3/503.3	0.07	0.48	0.06	(0.05)	0.20	1.21	60.00	0.23	0.25	0.56	0.07	0.11	0.40	3.55	60.00	0.00
	520.9/351.3	(0.07)	0.57	0.06	(0.20)	0.05	(1.22)	86.00	0.23	0.11	0.62	0.07	(0.02)	0.24	1.64	86.00	0.11
	520.9/593.1	(0.02)	0.56	0.06	(0.15)	0.10	(0.35)	76.00	0.72	0.19	0.56	0.06	0.06	0.31	2.96	76.00	0.00

AUROCs of 6 ions models (176.1, 353.7, 459.8, 503.3, 351.3, 593.1) discriminating inflammatory grades G2-G4 from G0-G1 were 0.840, 0.843, 0.694, 0.856, 0.902 and 0.868, respectively (Figure 4C-1). AUROCs distinguishing G3-G4 from G0-G2 were 0.886, 0.790, 0.731, 0.888, 0.887 and 0.787, respectively (Figure 4C-2). The results indicate that ion models based on 520.3 also had good sensitivity and specificity in discriminating liver inflammation grades of CHB patients.

### Detection of serum DAK protein in CHB patients

**Figure 5:**
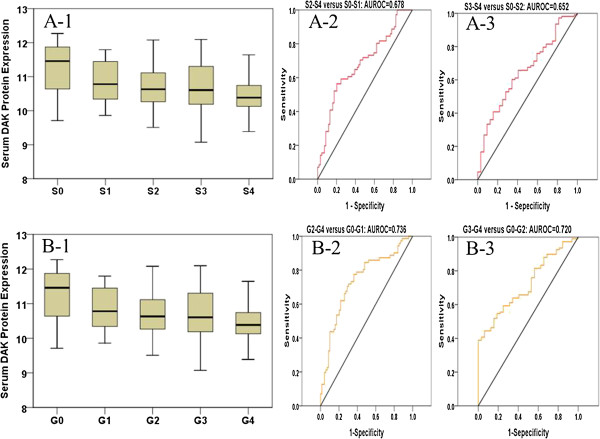
**Detection of serum DAK protein in CHB patients. A-1**: Serum DAK levels decrease with disease severity among groups classified by fibrosis degrees (S0-S4 subgroups) in 126 CHB patients. **A-2**: AUROC of DAK protein is 0.678 (S2-4 versus S0-1). **A-3**: AUROC of DAK protein is 0.652 (S3-4 versus S0-2). **B-1**: Serum DAK levels decrease with disease severity among groups classified by inflammatory grades (G0-G4 subgroups) in 126 CHB patients. **B-2**: AUROC of DAK protein is 0.736 (G2-4 versus G0-1). **B-3**: AUROC of DAK protein is 0.720 (G3-4 versus G0-2).

From all 126 patients, serum DAK protein was examined by ELISA. DAK levels mildly decreased with liver fibrosis and inflammation worsening (Figure 5A-1/B-1). When comparing fibrosis or inflammation subgroups, most of them had no statistically significant difference (Additional file 8: Table S5). The AUROC of DAK was 0.678 to distinguish S2-S4 from S0-S1 and 0.652 to distinguish S3-S4 from S0-S2 (Figure 5A-2/A-3). Further, AUROC of DAK was 0.736 to distinguish G2-G4 from G0-G1 and 0.720 to distinguish G3-G4 from G0-G2 (Figure 5B-2/B-3).

## Discussion

Native peptides are regarded as surrogate markers for protease activity in biological samples. Clinical peptidomics has been increasingly and widely used over the past few years, especially as potential biomarkers in cancer diagnosis [22]. Serum peptide quantitation represents challenge and technical difficulty to test and verify clinical significance. Due to recent technological advances, MRM has become a powerful method for defining the amount of a specific peptide sequence. It provides highly selective and sensitive quantification of candidate peptide fragments, whereby multiple assays can be performed in a single run. However, using these novel methods in liver disease has not yet been reported. In our study, we used LC-MS/MS-based peptidomics combined with MRM to identify serum peptides potentially useful as markers for liver fibrosis or inflammation progression in CHB patients. We robustly found serum ions levels of peptide m/z 520.3 decreased with more advanced fibrosis and inflammation, as compared to mild ones. Five from six diagnostic ions models of 520.3 (176.1, 353.7, 503.3, 351.3 and 593.1) were relatively accurate in discriminating degrees of liver fibrosis and inflammation, with one excepton, that is the ions 459.8 model. The new diagnostic models showed consistently high sensitivity and specificity, and markedly improved the discriminatory power to diagnose fibrosis and inflammation degrees S2/G2. Therefore, we suggest usage of serum ion model from peptide m/z 520.3 to guide timing of antiviral treatment and avoid liver biopsy.

The biomarker peptide m/z 520.3 was identified as peptide fragment from DAK, a bi-functional ATP-dependent dihydroxyacetone kinase. Human liver DAK characteristics and function were identified in 2005 [26]. DAK is a key enzyme that specifically inhibits MDA5-mediated innate antiviral signaling [27]. It is also associated with therapy outcome and responder classification with an accuracy of 85.7% for chronic hepatitis C patients [28]. So far, the molecular mechanisms by which a peptide fragment of DAK can reflect the degree of liver fibrosis and inflammation are unknown. The peptide maybe a marker of immune dysregulation associated with more pronounced hepatic inflammation and advanced liver disease.

We designed a serum peptidomic protocol suggesting a DAK peptide fragment as indicator of advanced fibrosis and inflammation in CHB patients, however, it is difficult to detect DAK peptide levels with routine clinical laboratory methods. Upon testing and verifying DAK protein expression in sera, results were consistent with serum peptidomics, however without statistical difference in CHB patients. It is possible that only a spliced form, which contains LLSKLSVLLLEKMG, but not full length DAK protein is an indicator for advanced fibrosis and inflammation. The potential mechanism behind needs further investigation. It may also be possible that MS based MRM is required for a clinical application as described for the DAK peptide. Difficulties then include depency on such MS instrument that is not available in most general hospital. The next step should be the development of a peptide-ELISA protocol to detect the DAK fragment LLSKLSVLLLEKMG in the serum [29,30].

Nonetheless, peptide m/z 520.3 of DAK as detected by serum peptidomics provides a novel non-invasive biomarker for evaluating degrees of liver fibrosis and inflammation in CHB patients that can be further developed.

## Abbreviations

CHB: Chronic hepatitis B; HBV: Hepatitis B virus; LC-MS/MS: Liquid chromatography combined with mass spectrometry; MRM: Multiple reaction monitoring; SPI: Scored peak intensity; SPAR: Summed peak areas ratio; APRI: Aspartate aminotransferase-to-platelet ratio index; DAK: Dihydroxyacetone kinase; AUROC: Area under receiver operating characteristic curve.

## Competing interests

The authors declare that no conflict of interest exists.

## Authors’ contributions

The work presented here was carried out in collaboration between all authors. M-YX, L-JZ and L-GL defined the research theme. M-YX and X-FJ designed methods and experiments, carried out the laboratory experiments, analyzed the data, interpreted the results and wrote the paper. YQ, H-LW and SD co-designed the dispersal and colonization experiments, and co-worked on associated data collection and their interpretation. X-PW co-designed experiments, discussed analyses, interpretation, and presentation. R-DZ and Z-HY provided the samples and co-worked on associated data collection and their interpretation. All authors have contributed to, seen and approved the manuscript.

## Supplementary Material

Additional file 1: Table S1 Clinical characteristics of enrolled patients in LC-MS/MS and MRM study groups. Click here for file

Additional file 2: Figure S1 Representative MS spectra of serum sample. Representative MS spectra of serum sample of stages (S0, S1, S3, S4, G0, G1, G3, G4) with retention time 52.5 ± 0.2 min and m/z range of 500-800. The corresponding amplified spectra with m/z rang 510-530 were shown on the right in which the DAK peptide (m/z 520.3) were labeled by arrow. Click here for file

Additional file 3: Table S2 Selected MRM transitions for the eleven analyzed differential peptides. Click here for file

Additional file 4: Figure S2 SPAR values and AUROCs of ions from peptides m/z 414.9 and m/z 735.5. The figure shows SPARs and AUROCs of 2 ions from m/z 414.9 (132.6, 226.6) and 4 ions from m/z 735.5 (215.3, 389.3, 460.3, 524.3) in 86 serum samples from CHB patients. Patients were classified into 5 groups according to fibrosis stages (S0: n = 13, S1: n = 22, S2: n = 20, S3: n = 16, S4: n = 15). A-1 and B-1: SPAR values of ions from m/z 414.9 and 735.5 display no statistically significant difference in groups (S1-S4 versus S0). A-2/3 and B-2/3: AUROCs of ions from m/z 414.9 are less than 0.5 and ions of m/z 735.5 are less than 0.8 (S2-S4 versus S0-1 or S3-4 versus S0-2). Click here for file

Additional file 5: Figure S3 Calibration curves for 735.5/389.3 of ESAT-6 and 6 analytes of peptide m/z 530.3. Seven calibration standards with concentrations of 6.25, 12.5, 25, 50, 100 and 200 ng/μl were used for MRM analysis. A: The correlation coefficients (r) of the calibration curves were >0.99 for 735.5/389.3 of ESAT-6, as determined by linear analysis. B: 6 ion pairs were analyzed including 4 son ions with double charge and 2 with single charge from 520.3. The correlation coefficients (r) of the calibration curves were >0.99 for all six analytes, as determined by linear analysis. All 6 ion pairs in the quantification range have an accuracy of 100 ± 20%. Click here for file

Additional file 6: Table S3 Precision and accuracy experiments of 520.3 ions. Click here for file

Additional file 7: Table S4. A. Stability of analyst 4°C for 24 h. B. Stability of analyst -80°C for 2 months. Click here for file

Additional file 8: Table S5 ELISA results comparison among fibrosis or inflammation subgroups. Click here for file
