# Supplementary figures and images for: Serum dihydroxyacetone kinase peptide m/z 520.3 as predictor of disease severity in patients with compensated chronic hepatitis B
Source: J Transl Med. 2013 Sep 27;11:234. doi: 10.1186/1479-5876-11-234 (PMC3851457; doi:10.1186/1479-5876-11-234)

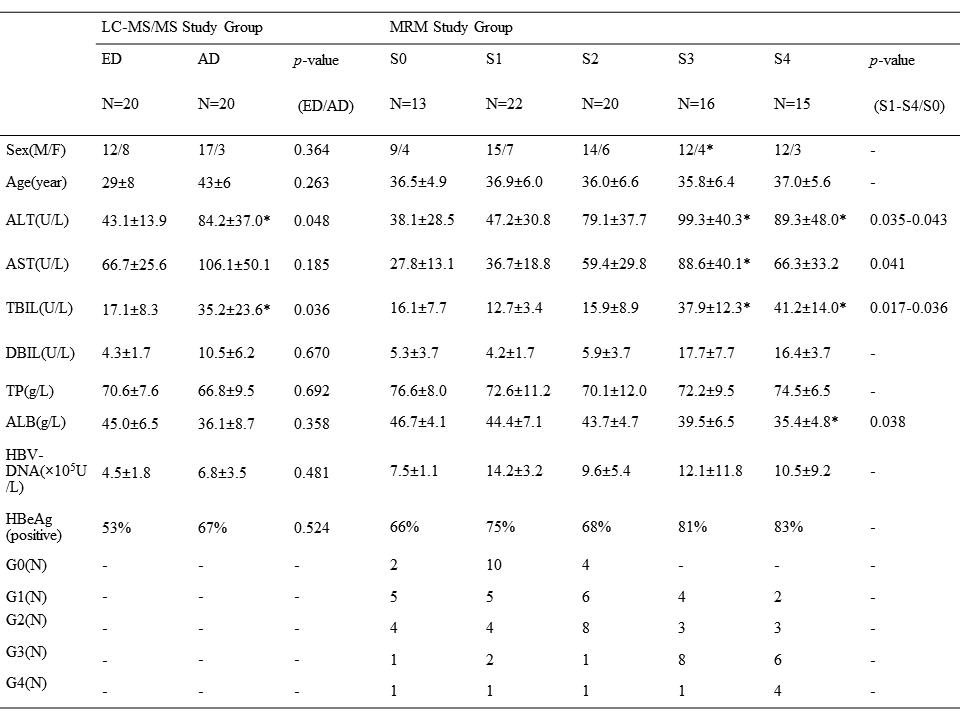

Supplement: Additional file 1: Table S1 — Clinical characteristics of enrolled patients in LC-MS/MS and MRM study groups. [file 1479-5876-11-234-S1.tiff]

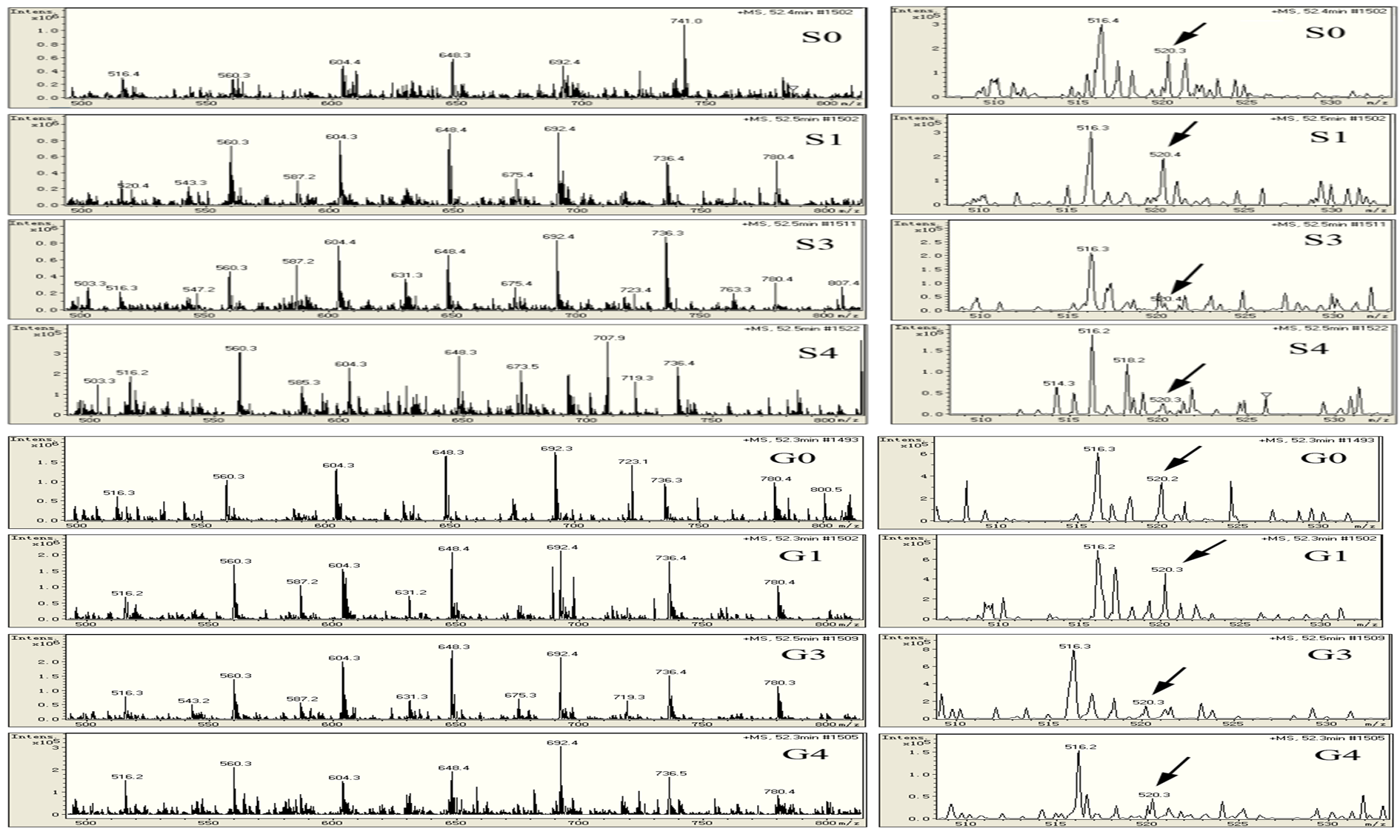

Supplement: Additional file 2: Figure S1 — Representative MS spectra of serum sample. Representative MS spectra of serum sample of stages (S0, S1, S3, S4, G0, G1, G3, G4) with retention time 52.5 ± 0.2 min and m/z range of 500-800. The corresponding amplified spectra with m/z rang 510-530 were shown on the right in which the DAK peptide (m/z 520.3) were labeled by arrow. [file 1479-5876-11-234-S2.tiff]

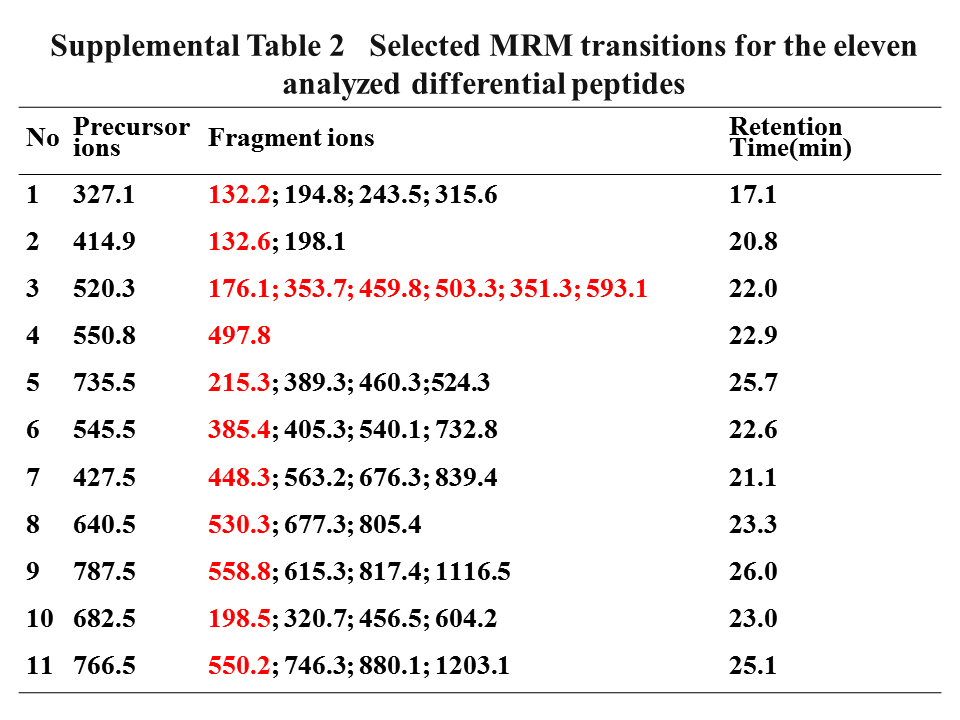

Supplement: Additional file 3: Table S2 — Selected MRM transitions for the eleven analyzed differential peptides. [file 1479-5876-11-234-S3.tiff]

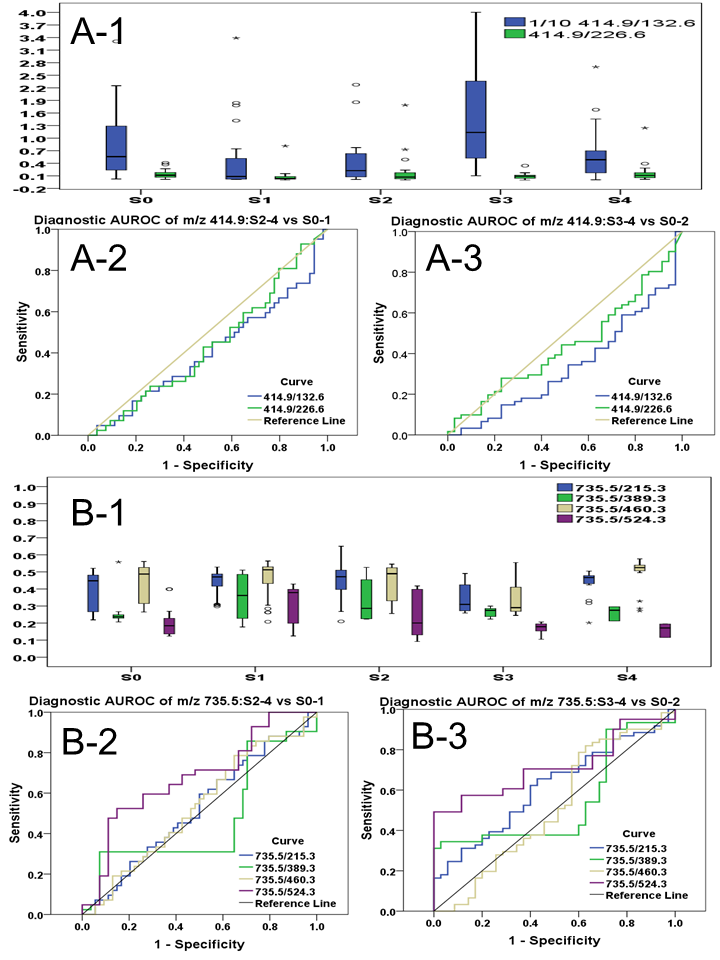

Supplement: Additional file 4: Figure S2 — SPAR values and AUROCs of ions from peptides m/z 414.9 and m/z 735.5. The figure shows SPARs and AUROCs of 2 ions from m/z 414.9 (132.6, 226.6) and 4 ions from m/z 735.5 (215.3, 389.3, 460.3, 524.3) in 86 serum samples from CHB patients. Patients were classified into 5 groups according to fibrosis stages (S0: n = 13, S1: n = 22, S2: n = 20, S3: n = 16, S4: n = 15). A-1 and B-1: SPAR values of ions from m/z 414.9 and 735.5 display no statistically significant difference in groups (S1-S4 versus S0). A-2/3 and B-2/3: AUROCs of ions from m/z 414.9 are less than 0.5 and ions of m/z 735.5 are less than 0.8 (S2-S4 versus S0-1 or S3-4 versus S0-2). [file 1479-5876-11-234-S4.tiff]

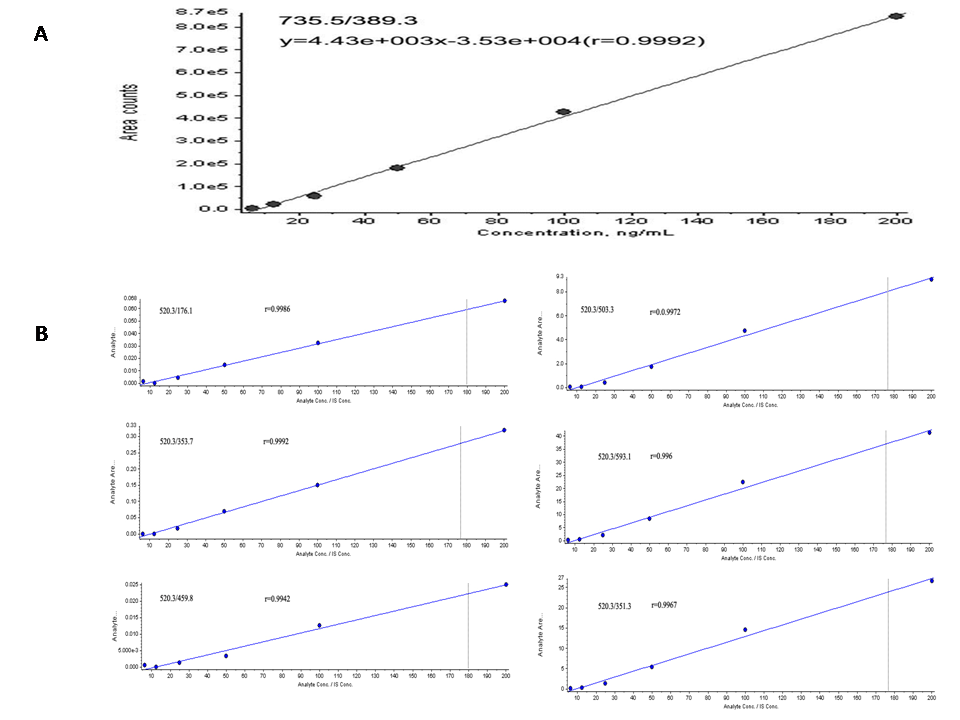

Supplement: Additional file 5: Figure S3 — Calibration curves for 735.5/389.3 of ESAT-6 and 6 analytes of peptide m/z 530.3. Seven calibration standards with concentrations of 6.25, 12.5, 25, 50, 100 and 200 ng/μl were used for MRM analysis. A: The correlation coefficients (r) of the calibration curves were >0.99 for 735.5/389.3 of ESAT-6, as determined by linear analysis. B: 6 ion pairs were analyzed including 4 son ions with double charge and 2 with single charge from 520.3. The correlation coefficients (r) of the calibration curves were >0.99 for all six analytes, as determined by linear analysis. All 6 ion pairs in the quantification range have an accuracy of 100 ± 20%. [file 1479-5876-11-234-S5.tiff]

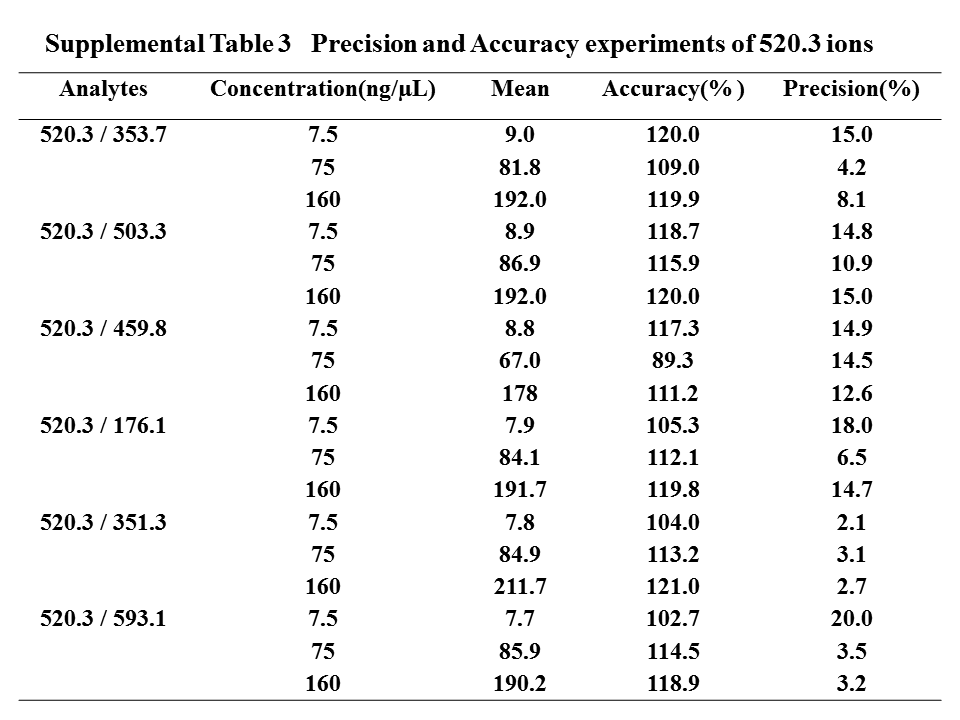

Supplement: Additional file 6: Table S3 — Precision and accuracy experiments of 520.3 ions. [file 1479-5876-11-234-S6.tiff]

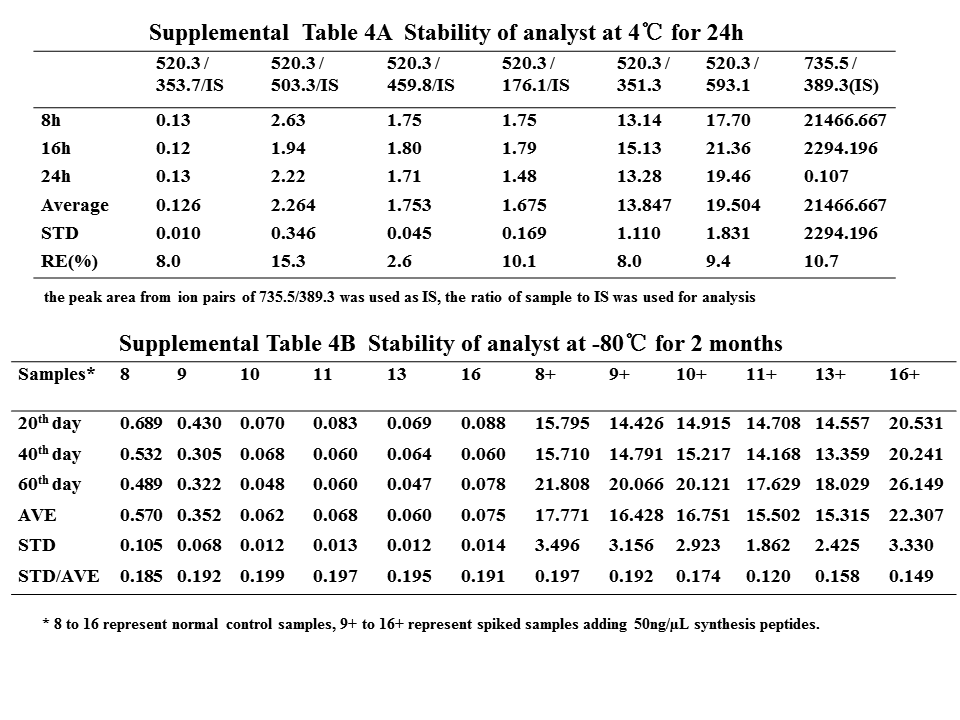

Supplement: Additional file 7: Table S4. — A. Stability of analyst 4°C for 24 h. B. Stability of analyst -80°C for 2 months. [file 1479-5876-11-234-S7.tiff]

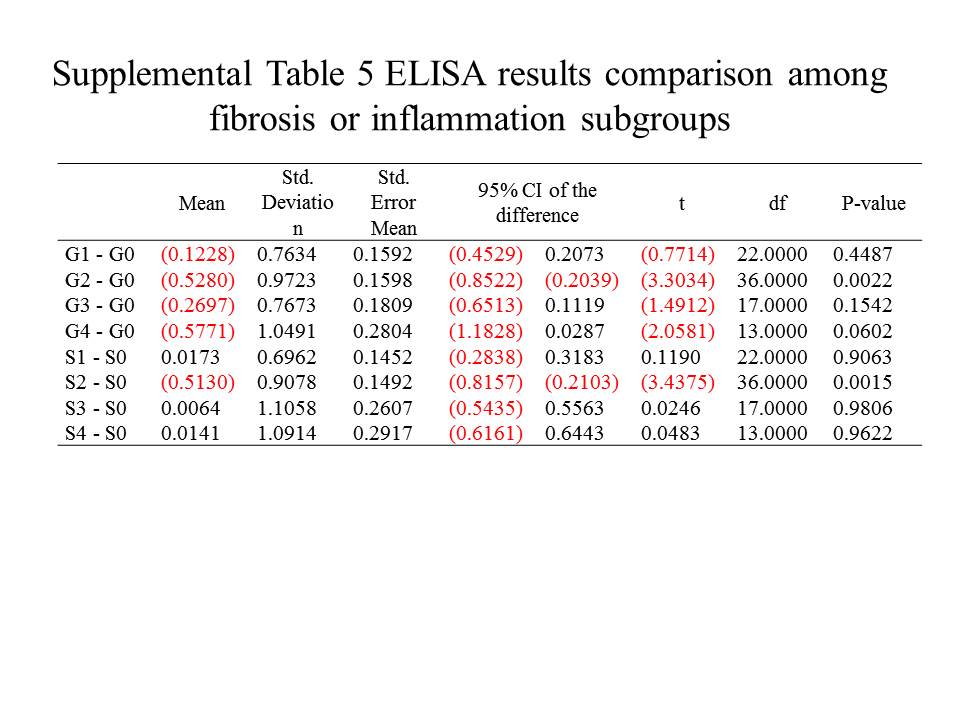

Supplement: Additional file 8: Table S5 — ELISA results comparison among fibrosis or inflammation subgroups. [file 1479-5876-11-234-S8.jpeg]
